# On the effectiveness of COVID-19 restrictions and lockdowns: Pan metron ariston

**DOI:** 10.1186/s12889-022-14177-7

**Published:** 2022-10-01

**Authors:** Leonidas Spiliopoulos

**Affiliations:** Max Planck Institute for Human Development, Center for Adaptive Rationality, 94 Lentzeallee, 14185 Berlin, Germany

**Keywords:** SARS-CoV-2, Non-pharmaceutical interventions, Case growth rate, Death growth rates, Simultaneous structural equations, Contact tracing, Vaccination

## Abstract

**Background:**

Early evaluations of the effectiveness of non-pharmaceutical intervention (NPI) mandates were constrained by the lack of empirical data, thereby also limiting model sophistication (e.g., models did not take into account the endogeneity of key variables).

**Methods:**

Observational analysis using a behavioral four-equation structural model that accounts for the endogeneity of many variables and correlated unobservable country characteristics. The dataset includes information from 132 countries from February 15, 2020, to April 14, 2021, with data on confirmed cases and deaths, mobility, vaccination and testing rates, and NPI stringency. The main outcomes of interest are the growth rates of confirmed cases and deaths.

**Results:**

There were strongly decreasing returns to more stringent NPI mandates. No additional impact was found for NPI mandates beyond a Stringency Index range of 51–60 for cases and 41–50 for deaths. A nonrestrictive policy of extensive and open testing constituted 51% [27% to 76%] of the impact on pandemic dynamics of the optimal NPIs. Reductions in mobility were found to increase, not decrease, both case $\left( -0.0417,\left[ -0.0578,-0.0256\right] ,p<0.001\right)$ and death growth rates $\left( -0.0162,\left[ -0.03,-0.002\right] ,p=0.025\right)$. More stringent restrictions on gatherings and international movement were found to be effective. Governments conditioned policy choices on recent pandemic dynamics, and were found to be more hesitant in de-escalating NPIs than they were in imposing them.

**Conclusion:**

At least 90% of the maximum effectiveness of NPI mandates is attainable with interventions associated with a Stringency Index in the range of 31–40, which impose minimal negative social externalities. This was significantly less than the average stringency level of implemented policies around the world during the same time period.

**Supplementary Information:**

The online version contains supplementary material available at 10.1186/s12889-022-14177-7.

## Background

**Fig. 1:**
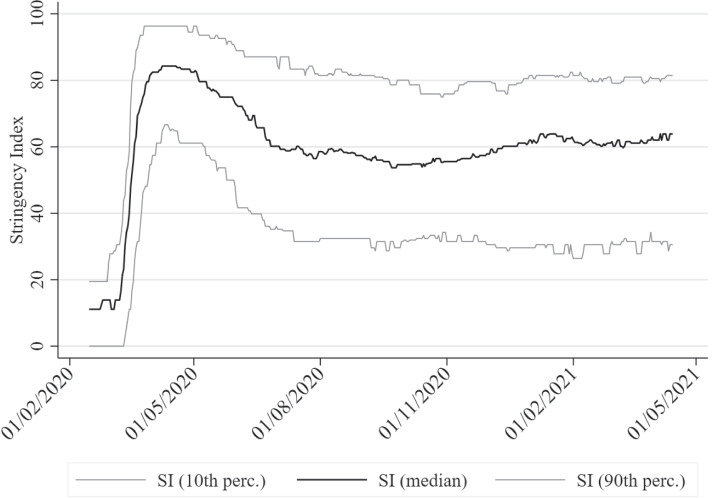
The time evolution of the Stringency Index across countries

The ancient Greek dictum *pan metron ariston*—everything in moderation—implies that extreme measures on either end of a spectrum are unlikely to be the best policy. During the COVID-19 pandemic, one extreme—no intervention—was ruled out by most governments early on. The other extreme, however—severe restrictions and even complete lockdowns—remained on the table: Severe lockdowns were imposed during the first wave of the pandemic, when uncertainty regarding the transmission and mortality of COVID-19 was high. This approach can be justified as a maxmin reaction in the face of uncertain events, minimizing the worst possible outcome. As we learn more about COVID-19 and uncertainty is reduced, better estimates of both the probability distribution and magnitude of possible outcomes can be inferred, allowing for a more nuanced approach aligned with an expected value calculation of costs and benefits. Nevertheless, severe *non-pharmaceutical interventions* (NPIs) remained in place in many countries despite the fact that governments and scientists were increasingly better informed. This can be seen in the evolution of the stringency of government-imposed NPIs (measured on the Stringency Index, SI, on a scale from 0 to 100) plotted in Figure 1, which includes the median SI value across countries and the 10th and 90th percentiles. Median SI peaked during the first wave in April 2020, reaching a maximum of 84. It then trended slowly downwards and leveled off to a range of 55–60, slowly trending up again as subsequent waves led to a resurgence of the pandemic. As of April 2021, while NPIs had not reached the peak levels of the first wave, the median SI across countries at the last datapoint (April 14, 2021) was still relatively high at 64 (10th perc.: 31; 90th: 81). Another important observation is that after the peak in April 2020, governments have followed increasingly heterogeneous NPIs, with the difference in the 10th and 90th percentiles increasing from roughly 30 SI points to around 50 SI points since late 2020.

The aim of this study is to exploit this divergence in government responses in order to more clearly identify the effects of the strictness of NPIs on the dynamics of the pandemic. Here I extend earlier work based on limited data from the first wave [1–4] and address certain econometric issues with prior analyses. Did the accumulated data from over a year support the continued imposition of stringent NPIs well into the second year of the pandemic? I test the whole spectrum of COVID policies in terms of varying degrees of severity to determine whether moderation is indeed the best approach. If so, it is crucial to be able to identify optimally moderate measures within the spectrum of policy responses because restrictions and lockdowns have an immense effect on economic and psychological well being that eventually translate into negative health outcomes [5–7]. Because these effects are complex and difficult to quantify, there is a general propensity to focus solely on the positive, immediate effects of NPIs. However, trade-offs are ubiquitous and we ignore them at our peril.

It should be noted that it may not always be possible to differentiate between the effects of announced NPI *mandates* and how effective NPIs could potentially be in an ideal world. That is, there may exist unobservable effects due to heterogeneous enforcement by governments or behavioral change by citizens across countries. Regarding stay-at-home mandates and other similar NPIs that aim to restrict the mobility of citizens, the inclusion of *observable* mobility data in the model will ameliorate this problem. In other cases where no observable behavioral variables are available, such effects are absorbed by country-dependent latent variables in the econometric model. A conservative reading of the findings, however, should consider that better enforcement of NPIs may have led to a more significant impact than estimated. On the other hand, it is possible that better enforcement (e.g., through extensive policing) may not be possible or desirable. In this case, the findings would coincide with the best possible *achievable* effects of NPI mandates, which is what should drive government policy from a practical perspective.

*Trade-offs in COVID-19 research.* Trade-offs also exist in the choice of pandemic research methodologies, both in terms of the type of model and the data it is estimated on. For example, models may be estimated on data at the national or subnational level. Working at the subnational level has the benefits of better controlling for country infection dynamics and avoiding possible issues arising from differences in pandemic accounting standards across countries [8]. However, since data at the national level is broader than subnational data in terms of country coverage, analyses at the national level may carry more external validity and are less likely to fall prey to overfitting due to small sample noise. Finally, the trade-off between aggregated (assuming homogeneity or pooled) or disaggregated (per country) modeling depends strongly on the amount and quality of the data.

The impact of policy interventions can be examined under the prism of three types of models, each with their own set of trade-offs: detailed models of epidemiological processes such as susceptible-infectious-removed (SIR)-based models) [9–11], agent-based modeling of transmission in a population (e.g., [12]), and reduced-form models that abstract away from epidemiological processes and agent interactions at the micro level by using simple econometric models that capture the effects of variables without inference of complex epidemiological parameters. Epidemiological models can be desirable because they directly model the underlying mechanisms, but they are prone to overfitting in the presence of scant or low-quality, noisy data. This leads to nonrobustness (see [13, 14]) since they require estimates of key parameters that are highly uncertain, and whose impact may reverberate significantly in highly nonlinear exponential growth models. No single approach dominates the rest, especially at the current stage of our understanding of the pandemic. Different approaches must be simultaneously pursued whilst acknowledging the limitations and advantages of each in an attempt to consolidate the findings.

As the literature on COVID-19 is sizable, I briefly survey the most similar studies to this manuscript, primarily those employing reduced-form equations. Early discourse was heavily influenced by the epidemiological modeling of the Imperial College COVID-19 Response Team [15], which concluded that lockdowns were a crucial and necessary strategy. A study using subnational data from six countries revealed that while not all NPIs have a significant impact, overall they significantly reduce infections [16]. However, it did not explicitly account for the stringency of individual NPIs. Dichotomizing the range of NPIs into less restrictive (e.g., social distancing, discouraging international and domestic travel, and banning large gatherings) and more restrictive (e.g., stay-home and business closure orders) reveals no evidence of additional gains associated with more restrictive NPIs in 10 countries at the subnational level [17]. Similarly, stringent lockdowns in 24 European countries in the first five months of 2020 did not significantly improve pandemic dynamics compared to less stringent lockdowns [18]. A similar finding emerged in a study using data from 108 countries [19]. This implies that NPIs may have a signalling effect that leads citizens to voluntarily change their behavior and that less stringent NPIs already signal strongly enough so that there is no room for improvement by implementing more stringent NPIs. The importance of voluntary behavior change is exemplified by the significant changes in mobility that occured before mandatory restrictions on movement came into effect [20, 21], and by the fact that mobility did not return to previous levels after lockdown restrictions were eased [22, 23]. The authors of a review of empirical studies [24] including some of the studies above concluded that the most effective interventions were (from most to least effective): school closings, workplace closings, business and venue closings, and public event bans. However, it should be noted that they often found conflicting results across studies, and that for many individual NPIs roughly only half the studies would find a statistically significant impact whereas the other half would not. For example, 58% of the studies found a significant impact of school closing on cases, 50% for public event bans and 57% for business and venue closing; however, 86% found that workplace closing was effective.

I seek to combine the advantages of reduced-form approaches whilst alleviating some of the drawbacks in their implementations to date. Specifically, earlier studies eschewed behavioral components and agents’ incentives, estimated a single regression disavowing variable endogeneity, and were estimated on datasets from the first few months of the pandemic without the benefit of data covering more recent developments (e.g., newer variants).

*The importance of behavioral models.* Models can be classified according to whether they are behavioral or not—that is, whether the behavioral responses of citizens and governments are allowed to vary endogenously or are assumed to be fixed. Agent-based models are behavioral, whereas standard SIR models are not. Reduced-form models typically estimate single-equation regressions of effects of NPI variables on either the confirmed case (and/or death) growth rate or mobility data, thereby implicitly assuming that these variables are exogenous. However, it reasonable to believe that mobility is dependent on the severity of the NPIs in place: The level of severity may mediate the impact of NPIs on COVID-19 dynamics. Furthermore, NPI stringency may also be endogenous if governments base their policy decisions on recent epidemiological data and trends (e.g., infection growth). Consequently, agents’ adaptive reactions to the situation also merit attention—both those of citizens [25] (e.g., mobility and precautionary and voluntary measures) and those of governments (e.g., the choice and timing of policies).

Behavioral components can be introduced into both epidemiological and reduced-form models. The former is significantly more common than the latter, which I pursue in this study. Incorporating behavioral components in SIR models leads to very different long-run predictions than those made by models based on standard nonbehavioral SIRs [26]. A common finding is that the system tends to an equilibrium reproduction rate of 1 [27], sometimes with oscillations if behavioral components operate with lags [28, 29]. These studies highlight that it is important to model the evolution of behavioral responses, as they can lead to important qualitative, not just quantitative, changes in pandemic dynamics. The canonical behavioral response depends on the perceived risk of infection and severity; for instance, consumers changed their shopping habits predominantly of their own volition, not due to legal restrictions [30]. Behavioral incentives can have a stabilizing effect on the infection growth rate; the higher the infection growth rate, the more likely citizens are to adjust their behavior to decrease the chance of infection. However, it is important to bear in mind that individual behavior may become more reckless as risks are mitigated (see the literature on risk compensation [31]). For example, as the pandemic starts to wane, citizens may adopt more risky practices, thereby slowing the decrease in the infection rate. Similarly, extensive testing and vaccinations may elicit adverse behavioral responses if citizens believe they are less likely to contract COVID-19 and infect others.

*The importance of beliefs and expectations.* Unobservable variables such as culture [32] may jointly influence key variables that are typically assumed to be exogenously determined. For example, low social capital and trust in government may affect case growth rates both directly and indirectly. Suppose that low-trust countries also tend to have inadequate public health systems. This will have a direct impact on case and death growth rates due to inadequate health care, but it may also have an indirect effect: Governments of these countries, knowing that the health care system is weak and intensive care units are scarce, may impose more stringent NPIs compared to countries with better health systems in a attempt to prevent intensive care units from filling to capacity. Citizens’ and governments’ expectations and beliefs can introduce further endogeneity. Citizens’ beliefs about the quality of governance may shape their behavior and impact the effectiveness of NPIs [33]. Low trust between citizens and government institutions may lead citizens to view their government’s portrayal of the situation with skepticism, and may encourage governments to impose stricter restrictions in anticipation of its citizens exhibiting a weaker behavioral response.

*Simultaneously modeling endogeneity and unobservable variables, behavioral incentives, and beliefs.* Acknowledging that confirmed case and death growth rates, mobility, and government policies are endogenous requires a system of four equations to fully model these interactions and avoid biases resulting from simpler regressions that implicitly impose exogeneity. I complement the literature by employing econometric procedures, namely structural multi-equation modeling including latent variables capturing the effects of unobservable determinants, to improve upon the external validity of prior analyses. This system of equations can be viewed through the lens of game theory—that is, modeling agents who react to each other’s strategies and their expectations thereof. I propose an admittedly rudimentary model of the adaptive interactions of three agents: the SARS-CoV-2 virus, citizens, and governments.^1^ My approach complements existing studies by investigating a large number of countries. This approach has the advantage of pooling estimates for robustness, but also disadvantages such as the assumption of homogeneity across countries and the need to focus on aggregated measures of key endogenous variables—rather than their individual components [1]—in order to avoid a computationally intractable and unidentifiable system of equations. A central issue is to examine whether there are decreasing returns as NPIs increase in severity, and, if so, to estimate the optimal level of severity of government policies. This is critical not only for future COVID-19 variants and other pandemics, but also currently, since less strict NPIs may also be valuable during the vaccination phase [34].

## Methods

National-level data from 132 countries covering the period from February 15, 2020, to April 14, 2021, was compiled, extending previous analyses to data including the appearance and spread of the B.1.1.7 and B.1.351 variants detected in late 2020, and more recently discovered variants such as P.1. Confirmed case and death counts, vaccinations, and tests, were download from the COVID-19 Data Hub [35, 36], which also included the implementation of NPIs as measured by the composite SI sourced originally from the Oxford COVID-19 Government Response Tracker [37]. Mobility data from the Google Community Mobility Report [38] was merged with the data file from the COVID-19 Data Hub.

The growth rate of confirmed cases (deaths) was calculated as the log difference in the cumulative confirmed cases (deaths) for two consecutive days multiplied by 100 (i.e., they can be interpreted as approximate percentage growth rates). The summary statistics for growth in confirmed cases are: number of observations = 53,279, mean = 2.74%, stdev. = 8.87%; the summary statistics for growth in confirmed deaths are: number of observations = 49,366, mean = 1.997%, stdev. = 6.84%. To allow for a nonlinear relationship between the SI and case/death growth rate, a semiparametric approach was implemented by subdividing the SI (ranging continuously from 0 to 100) into a baseline of no restrictions (0) and the deciles (1–10, 11–20, ..., 91–100). The set of nonrestrictive policies examined are: the testing policy (TP), which takes on levels (*l*) of 0 (no testing) through 3 (open testing); the contact tracing (CT) policy, which takes on levels of 0 through 2; the proportion of the population tested per day (TPop); and the cumulative percentage of the number of vaccinations compared to the country’s population (V)—this can be greater than 100% because some vaccines require more than one dose.

**Fig. 2:**
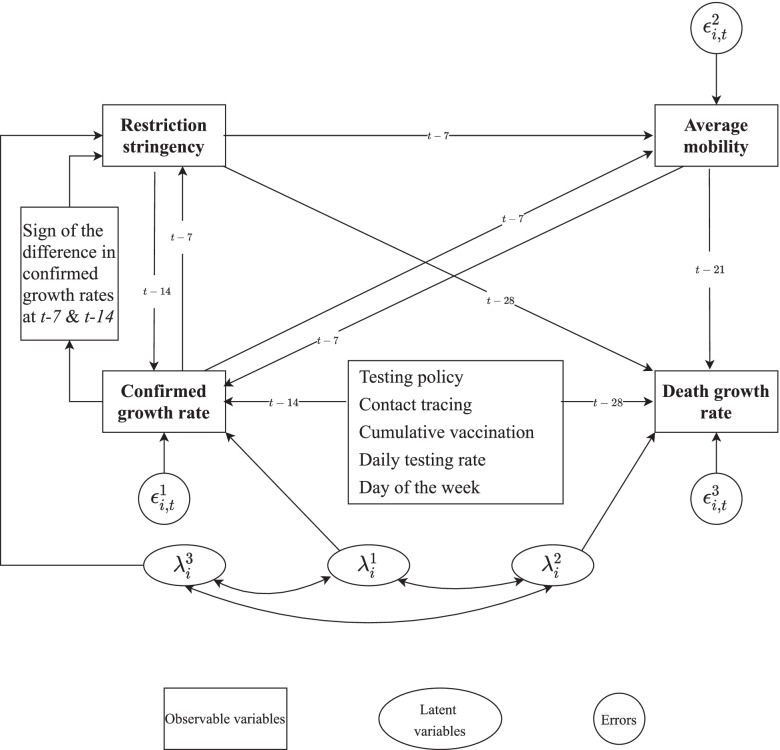
A four-equation structural model of COVID-19 dynamics

I address the issues regarding the previous modeling as identified above by simultaneously estimating four generalized structural equations to model the complex inter-relationships between variables (see Figure 2 for a graphical representation of the causal structure and Eqs. 1–4, which define the econometric model). The dependent variables for each equation are: (Eq. 1) the growth rate of confirmed cases ($\dot{C}$), (Eq. 2) the growth rate of confirmed deaths ($\dot{D}$), (Eq. 3) the seven-day moving average of mobility ($M_{i,t}$), and (Eq. 4) $p\left( SI_{i,t}=l\right)$ is the probability that the SI level belongs to the *l*th decile; note that $\varLambda$ is the logistic function. Below I describe the two variables $M_{i,t}$ and $S_{i,t}^{l}$ (growth rates of confirmed cases and deaths are self-explanatory).

$M_{i,t}$ denotes the seven-day moving average of the percentage change in mobility (compared to the pre-COVID baseline for each country)—this was constructed by an equal weighting of three individual variables separately measuring mobility towards transit stations, workplaces, and groceries and pharmacies. Since this variable is normalized at a different baseline per country, additional random effects at the national level were not implemented in Eq. 3, in contrast to the other equations.

1 $$\begin{aligned}{\dot C}_{i,t}&=\alpha^1+\beta_1^cM_{i,t-7}+\sum_{l=1,...,10}\beta_{2,l}^cSI_{i,t-14}^l+\sum_{l=1,2,3}\beta_{3,l}^cTP_{i,t-14,l}^l+\sum_{l=1,2}\beta_{4,l}^cCT_{i,t-14,l}^l\\&\quad+\beta_5^cTPop_{i,t-14}+\beta_6^cV_{i,t-14}+\sum_{l=1,...,6}\beta_{7,l}^cW_t^l+\lambda_i^1+\epsilon_{i,t}^1\end{aligned}$$

2 $$\begin{aligned}{\dot D}_{i,t}&=\alpha^2+\beta_1^dM_{i,t-21}+\sum_{l=1,...,10}\beta_{2,l}^dSI_{i,t-28}^l+\sum_{l=1,2,3}\beta_{3,l}^dTP_{i,t-28,l}^l+\sum_{l=1,2}\beta_{4,l}^dCT_{i,t-28,l}^l\\&\quad+\beta_5^dTPop_{i,t-28}+\beta_6^dV_{i,t-28}+\sum_{l=1,...,6}\beta_{7,l}^dW_t^l+\lambda_i^2+\epsilon_{i,t}^2\end{aligned}$$

3 $$M_{i,t}=\alpha ^{3}+\sum \limits _{l=1,...,10}\gamma _{1,l}SI_{i,t-7}^{l}+\gamma _{2}\dot{C}_{i,t-7}+\epsilon _{i,t}^{3}$$

4 $$\begin{aligned} p\left( SI_{i,t}=l\right)&=\Lambda \left( -\alpha _{l}^{4}+\delta _{1}\dot{C}_{i,t-7}+\delta _{2}I\left[ \dot{C}_{i,t-7}-\dot{C}_{i,t-14}\right] +\lambda _{i}^{4}\right) \\&\quad-\Lambda \left( -\alpha _{l-1}^{4}+\delta _{1}\dot{C}_{i,t-7}+\delta _{2}I\left[ \dot{C}_{i,t-7}-\dot{C}_{i,t-14}\right] +\lambda _{i}^{4}\right) \\ \end{aligned}$$

$$\begin{aligned}&\left( \lambda _{i}^{1},\lambda _{i}^{2},\lambda _{i}^{4}\right) \sim \mathcal {N}\left( \mathbf {0},{\varvec{\Sigma }_{\varvec{\lambda }}}\right) ,\varvec{\Sigma }_{\varvec{\lambda }}= \left[ \begin{array}{ccc} \sigma _{\lambda ,1}^{2} &{} \sigma _{\lambda ,12} &{} \sigma _{\lambda ,14}\\ \sigma _{\lambda ,12} &{} \sigma _{\lambda ,2}^{2} &{} \sigma _{\lambda ,24}\\ \sigma _{\lambda ,14} &{} \sigma _{\lambda ,24} &{} \sigma _{\lambda ,4}^{2} \end{array}\right]  \\&\left( \epsilon _{i}^{1},\epsilon _{i}^{2},\epsilon _{i}^{3}\right) \sim \mathcal {N}\left( \mathbf {0},\varvec{\Sigma }_{\varvec{\epsilon }}\right) ,\varvec{\Sigma }_{\epsilon }= \left[ \begin{array}{ccc} \sigma _{\epsilon ,1}^{2} &{} 0 &{} 0\\ 0 &{} \sigma _{\epsilon ,2}^{2} &{} 0\\ 0 &{} 0 &{} \sigma _{\epsilon ,3}^{2} \end{array}\right] \end{aligned}$$

Note that in Eq. 4, $SI_{i,t}$ is an ordered variable of the SI ranging in *levels* from 0 to 10, whereas in Eq. 1, $SI_{i,t}^{l}$ enters as a set of *l* dummy variables. If $\delta _{1}>0$ then governments respond to higher (lagged) case growth rates $\left( \dot{C}_{i,t-7}\right)$ by increasing the SI of their mandated NPIs. An indicator function $I\left[ \dot{C}_{i,t-7}-\dot{C}_{i,t-14}\right]$ captures government hysteresis or path dependence, which is equal to 1 if the case growth rate has been declining or zero if it has been increasing. The hypothesis is that governments are slower at scaling back stringent NPIs as the pandemic threat recedes than they are in implementing them during outbreaks. If the estimated coefficient $\delta _{2}>0$, for the same level of case growth, governments will on average be more likely to impose more stringent NPIs if the case growth trend is negative than if it is positive.

National-level unobservable characteristics were modeled using unique random effects denoted by $\lambda _{i}^{e}$ (latent variables) where *e* indexes the Eqs. (1, 2, or 4), allowing for covariance between all the random effects to capture the joint impact of unobservable country characteristics. Fixed effects for the impact of the day of the week on case and death growth rates are denoted by $W_{t}^{l}$.

Relationships between the case (and death) growth rates and other variables incorporated lags to capture the approximate delays in symptom onset and case confirmation and the timing of exposure to the virus [39]. The lags for the growth rate in deaths are 14 days longer than those for cases to reflect the fact that on average deaths occur at a much later time after infection (e.g., after lengthy hospitalizations). The exact determination of the lags is not critical, as there is a high degree of autocorrelation of the key lagged variables. To simplify the model, all lags and moving averages were multiples of seven days to remove the effects of systematic daily variations. Cluster-robust standard errors were employed to account for heteroskedasticity and within-panel dependence of errors.

The initial date used in the estimation varied by country as it was to set to the first day with a confirmed case. Any missing datapoints for the confirmed cases and deaths, number of tests, and number of vaccinations at time *t* were set equal to the value at time $t-1;$ this was done on the raw data of these variables, which were coded as cumulative sums until time *t*.

Several robustness tests were performed by estimating the same four structural equations under alternative assumptions. The first implicit assumption in Eqs. 1–4 tested for robustness is that the system’s coefficients are constant across the different COVID-19 infection waves and independent of the emergence of different SARS-CoV-2 variants. We follow a proposed classification of the first three COVID-19 waves [40], where the first wave began approximately in March 2020, the second wave in July 2020, and the third wave in January 2021. The model was estimated again with data including only: a) the first wave with a cutoff of June 30 (see Supplementary Table 7 for the estimation results) and b) the first and second waves with a cutoff of December 31, 2020 (see Supplementary Table 8). These roughly correspond to the following variants: the first wave mostly captures the Beta variant, the combination of the first and second waves capture the Beta and Alpha variants, whereas the complete dataset (reported in the main text) includes the Beta, Alpha and Gamma variants. The classification into these three waves (and the predominance of the corresponding variants) should be viewed as a necessary approximation to aid the modelling, as the exact dates vary across countries. Although this rudimentary classification is sufficient for the purposes of robustness tests, the results should not be viewed as decisive in assessing the possible differential impact of SARS-CoV-2 variants. Finally, the robustness of the decision to employ semi-parametric estimation by binning the SI into deciles was tested by estimating the model with the SI binned into quintiles instead (0, 1–20, 21–40, 41–60, 61–80, 81–100); the estimation results can be found in Supplementary Table 6. Across all the robustness tests, the main qualitative findings regarding the effectiveness of NPIs matched those of the model presented below in the main text (namely that the effectiveness of NPIs plateaued at moderate levels of stringency), and the variation in the magnitude of the impact across the robustness tests, was of one order of magnitude smaller than the impact reported for the main estimation.

The usual disclaimers hold regarding the use of observational data and the causal assumptions embedded in the chosen structural equations and variable relationships.

## Results

**Fig. 3:**
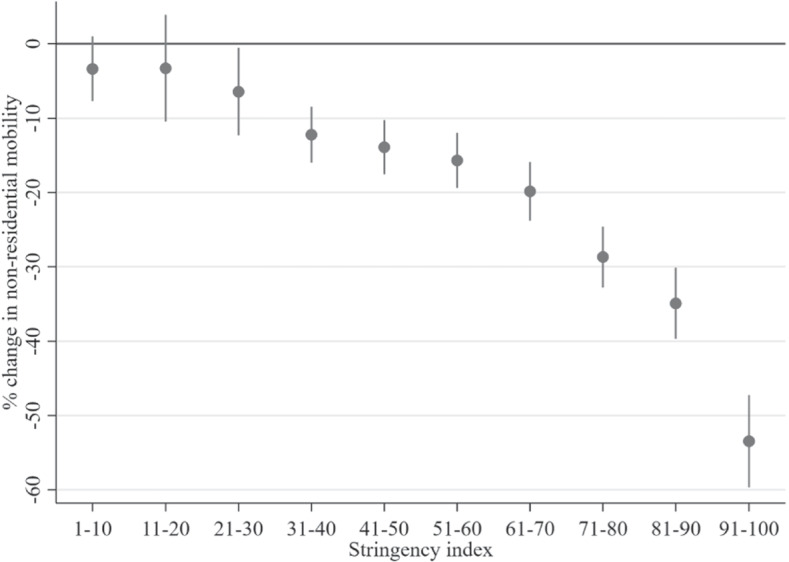
Impact of policy Stringency Index on the percentage change in nonresidential mobility $\left( \gamma _{1,l}\right)$

**Fig. 4:**
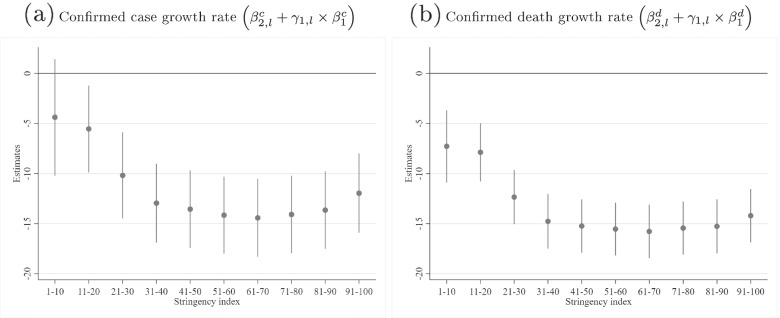
Total effect of the Stringency Index level

**Fig. 5:**
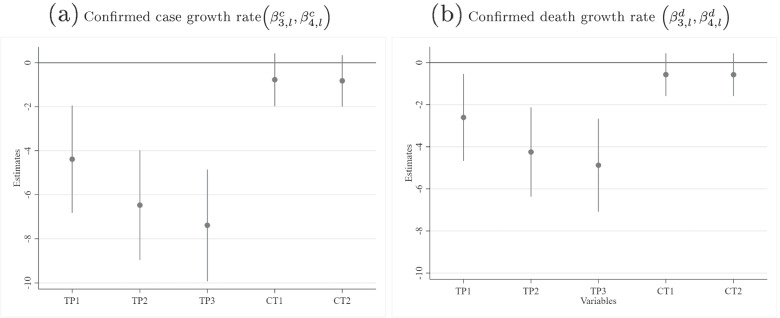
Estimated coefficients across levels of testing policy (TP*l*) and contact tracing (CT*l*), where *l*=level

Detailed regression results can be found in Table 1 and Supplementary Table 2; Figures 3, 4 and 5 present the main estimates graphically.

**Table 1:** Generalized structural equation model of $\dot{C}_{i,t}$, $\dot{D}_{i,t},M_{i,t}$, and $SI_{i,t}$

$\dot{C}_{i,t}$		$\dot{D}_{i,t}$		$M_{i,t}$		$SI_{i,t}$	
$M_{i,t-7}$	-0.0417***	$M_{i,t-21}$	-0.0162*				
	[-0.0578,-0.0256]		[-0.0304,-0.00199]				
$SI_{i,t-14}$		$SI_{i,t-28}$		$SI_{i,t-7}$			
1–10	-4.522		-7.336***	1–10	-3.346		
	[-10.40,1.350]		[-10.97,-3.701]		[-7.675,0.983]		
11–20	-5.681*		-7.938***	11–20	-3.264		
	[-10.01,-1.352]		[-10.85,-5.022]		[-10.44,3.910]		
21–30	-10.44***		-12.45***	21–30	-6.421*		
	[-14.73,-6.153]		[-15.16,-9.734]		[-12.29,-0.550]		
31–40	-13.45***		-14.96***	31–40	-12.21***		
	[-17.42,-9.487]		[-17.71,-12.22]		[-15.98,-8.438]		
41–50	-14.13***		-15.46***	41–50	-13.90***		
	[-18.04,-10.22]		[-18.14,-12.79]		[-17.54,-10.26]		
51–60	-14.81***		-15.80***	51–60	-15.68***		
	[-18.70,-10.92]		[-18.45,-13.14]		[-19.40,-11.96]		
61–70	-15.25***		-16.10***	61–70	-19.84***		
	[-19.18,-11.33]		[-18.80,-13.40]		[-23.80,-15.88]		
71–80	-15.28***		-15.91***	71–80	-28.68***		
	[-19.19,-11.36]		[-18.59,-13.22]		[-32.76,-24.59]		
81–90	-15.10***		-15.83***	81–90	-34.90***		
	[-19.04,-11.17]		[-18.57,-13.10]		[-39.67,-30.13]		
91–100	-14.19***		-15.07***	91–100	-53.46***		
	[-18.23,-10.15]		[-17.84,-12.30]		[-59.67,-47.25]		
${Tpop_{i,t-14}}$	-0.184	${Tpop_{i,t-28}}$	-0.126	$\dot{C}_{i,t-7}$	-0.214***	$\dot{C}_{i,t-7}$	0.0484***
	[-0.404,0.0355]		[-0.315,0.0629]		[-0.255,-0.174]		[0.0354,0.0613]
$V_{i,t-14}$	-0.0170*	$V_{i,t-28}$	-0.0187*			$I\left[ \dot{C}_{i,t-7}\right.$	0.531***
	[-0.0316,-0.00242]		[-0.0352,-0.00230]			$\left. -\dot{C}_{i,t-14}\right]$	[0.453,0.609]
$TP_{i,t-14}(1)$	-4.385***	$TP_{i,t-28}(1)$	-2.603*				
	[-6.824,-1.946]		[-4.671,-0.535]				
$TP_{i,t-14}(2)$	-6.474***	$TP_{i,t-28}(2)$	-4.247***				
	[-8.964,-3.983]		[-6.369,-2.125]				
$TP_{i,t-14}(3)$	-7.390***	$TP_{i,t-28}(3)$	-4.875***				
	[-9.926,-4.854]		[-7.086,-2.664]				
$CT_{i,t-14}(1)$	-0.77	$CT_{i,t-28}(1)$	-0.567				
	[-1.973,0.434]		[-1.584,0.450]				
$CT_{i,t-14}(2)$	-0.82	$CT_{i,t-28}(2)$	-0.572				
	[-1.986,0.345]		[-1.586,0.442]				
$\alpha ^{1}$	21.96***	$\alpha ^{2}$	20.71***	$\alpha ^{3}$	3.328*		
	[17.92,25.99]		[18.30,23.13]		[0.0958,6.560]		
$\sigma _{\lambda ,1}^{2}$	4.299***	$\sigma _{\lambda ,2}^{2}$	2.870***			$\sigma _{\lambda ,4}^{2}$	3.240***
	[3.312,5.287]		[2.076,3.665]				[2.350,4.130]
$\sigma _{\epsilon ,1}^{2}$	22.22***	$\sigma _{\epsilon ,2}^{2}$	33.72***	$\sigma _{\epsilon ,3}^{2}$	200.4***		
	[17.82,26.62]		[29.39,38.04]		[160.2,240.5]		

$$\sigma _{\lambda ,12}=3.429^{{*}{*}{*}}\ [2.683,4.176],\ \sigma _{\lambda ,14}=2.478^{{*}{*}{*}}\ [1.798,3.158],\ \sigma _{\lambda ,24}=2.070^{{*}{*}{*}} [1.479,2.661]$$

$$\lbrack95\%\mathrm{CI}\rbrack,\;{}^\ast\;p<0.05,\;{}^{\ast\ast}p<0.01,\;{}^{\ast\ast\ast}p<0.001$$

*Highly correlated unobservable variables influence both government policy and confirmed case and death growth rates* The estimated covariances (and associated correlations) between the national-level latent variables in Eqs. 1, 2, and 4 were all positive and significantly different from zero at the 0.001% level: $\rho _{\lambda ,12}$=0.98 [95% CI: 0.95,1.00], $\rho _{\lambda ,14}$=0.66 [0.54,0.79], $\rho _{\lambda ,24}$=0.68 [0.56,0.80]. This validates the hypothesis that significant unobservable variables may simultaneously influence case and death growth rates and the severity of government policies. Ignoring these relationships by estimating a single regression of case or death growth rates, as is commonly done in the literature, would have led to biased estimates.

*Adaptive expectations of the risk of infection impact nonresidential mobility* Citizens reacted to increases in the seven-day lag in growth rates of confirmed cases by reducing mobility. This is consistent with a theory of citizens forming adaptive expectations about the severity of the pandemic at any point in time and the risk of contraction based on the data. The impact of the daily growth in confirmed cases is $\hat{\gamma }_{2}=-0.214$ [-0.255,-0.174]. Putting the effect size into perspective, an increase in the case rate of 1 percentage point would result on average in a relatively weak reduction in mobility of 0.21 percentage points compared to the baseline.

*The indirect links between NPIs and confirmed case/death growth rates* The indirect links between NPI mandates and confirmed case/death growth rates consist of two components: for cases, ($SI\rightarrow M)$ and ($M\rightarrow \dot{C})$, and for deaths, ($SI\rightarrow M)$ and ($M\rightarrow \dot{D})$. Below, I will present evidence that nonresidential mobility mediated the relationship between NPI stringency and case/death growth rates. I begin by reporting the evidence regarding the $SI\rightarrow M$ component, which is common to both instances.

*Higher NPI severity restricts nonresidential mobility* Nonresidential mobility is clearly impacted by the SI (see the estimates of $\gamma _{l,t}$ in Table 1 and Figure 3), as the null hypothesis that all SI dummy variable coefficients are equal to zero was rejected ($\chi ^{2}\left( 10\right) =428.38,p<0.0001$). Furthermore, the absolute value of the effect size was monotonically increasing in the 10 SI ranges, i.e., higher stringency led to reduced mobility.

*Lower nonresidential mobility increases case and death growth rates* The theoretical motivation behind restricting mobility through lockdown measures is that restricting it will reduce the number of social interactions, thereby reducing transmission and infection. However, a reduction in nonresidential mobility necessarily entails an increase in the time spent within residences—indeed, these two effects are highly negatively correlated in the Google mobility data, where the median correlation within panels is -0.89. Consequently, reductions in nonresidential mobility may have a detrimental effect on case/death growth rates; although transmission outside the home may be reduced, within-household transmission may be enhanced as people spend increasingly more time with cohabitants in the confines of a closed space [41]. Since the two effects work in opposite directions, the question of whether restricting nonresidential mobility reduces case/death growth rates must be resolved empirically. The finding that the impact of $M_{i,t-7}$ was significantly negative for cases, $\hat{\beta }_{1}^{c}=-0.0417$ $\left( \left[ -0.0578,-0.0256\right] ,p<0.001\right)$, and of $M_{i,t-21}$ was significantly negative for deaths $\hat{\beta }_{1}^{d}=-0.0162$ $\left( \left[ -0.03,-0.002\right] ,p=0.025\right)$, supports the hypothesis that the benefits of reduced nonresidential mobility are more than outweighed by the detrimental effects of increased within-household transmission (conditioned on the stringency of government policies). The effect size is moderate, as a 10 percentage point decrease in mobility leads to an increase of 0.4 percentage points in the case growth rate.

*The direct link between SI and case/death growth rates* The null hypothesis that NPI mandates had no direct impact on case growth rates was rejected, as the set of $\beta _{2,l}^{c}$ estimates are all equal not significantly different from zero ($\chi ^{2}\left( 10\right) =137.28,p<0.0001$). Furthermore, there was evidence of a nonlinear relationship between NPI stringency and case/death growth rates, with increasingly stringent restrictions offering strongly decreasing returns (see Table 1; the conclusions are similar for death growth rates).

*The total effect of NPI stringency on case/death growth rates and the optimal level of stringency* The total effect of the stringency of NPI mandates on case growth rates can be computed by adding the direct path $\beta _{2,l}$ and the indirect path $\gamma _{1,l}\times \beta _{1}$ for each stringency level of *l*. This combined effect of stringency on $\dot{C}_{i,t}$ and $\dot{D}_{i,t}$ is documented in Supplementary Tables 2 and 4, and presented graphically in Figure 4.

The maximum impact of NPI stringency on case growth is observed for the SI range of 61–70. I tested the difference in effectiveness of all other levels against the most effective range, 61–70, correcting for multiple comparisons using the Sidak correction.^2^ There was no difference in effectiveness for the ranges 51–60 and 71–80 compared to 61–70 (see Supplementary Table 3 for the test statistics). The ranges 81–90 and 91–100 were significantly less effective than the range 61–70. Consequently, there are no further gains to be achieved beyond the SI range of 51–60. The socially optimal SI range, however, must account not only for the positive effects of NPIs, but also for the significant impact on physical and mental health [42–48] and economic costs that result from restrictions (for example, see [5–7]). While this would require a full cost–benefit analysis [49–52] that is beyond the scope of this paper, it is possible to derive the approximate upper bound of the socially optimal SI level with a single assumption about the cost profiles of different SI levels: that the costs are monotonically increasing in the SI level. Consequently, without the need to quantify costs, I conclude that the upper bound of the socially optimal SI level, SI*, is 51–60—that is, the minimum SI range that is not significantly different from the maximum effect at 61–70.

Quantifying the exact costs of NPIs is an important endeavour, but one that is fraught with difficulties, such as converting non-economic outcomes into monetary terms. Furthermore, doing so would require multiple assumptions about highly uncertain possible effects, many in the distant future. By contrast, this upper bound on SI* is extremely robust, albeit less informative. Although the derivation of SI* is based on statistical significance, it is possible that lower SI levels may be statistically significantly different from SI*, but that the difference in the effect size is practically of little importance. Indeed, the ratio of the effect sizes of an SI range of 31–40 relative to the average of the 51–80 range, is 91% [$85\%,97\%$], that is, the former is 91% as effective as the latter. The relative effectiveness of the even laxer 21–30 range is 72% [$57\%,86\%].$ If the costs of NPIs increase quickly with NPI stringency, it is conceivable that moderately severe policy responses in the 31–40 SI range (and possibly even 21–30) may in fact be close to the socially optimal SI* (arising from a full cost–benefit analysis), as it achieves 91% effectiveness without accounting for costs.

Similarly, the maximum effectiveness on death growth rates was also observed in the SI range of 61–70. I tested the difference in effectiveness of all other levels against this range and found no significant increase in effectiveness for levels beyond 41–50 (see Supplementary Table 5). An SI range of 31–40 achieved 93% [$87\%,98\%$] of the effectiveness of the average of the 41–90 range; the laxer 21–30 range achieved 73% [$58\%,88\%].$

Finally, while the SI aggregates individual NPI policies, examining the median values of the individual policies in the dataset for each SI range can also be informative (see Table 2). Note that in the 31–40 SI range, which achieves at least 90% of the maximum impact, the median policies do not include any restrictions on transport and internal movement, nor do they include stay-home restrictions. Furthermore, closing schools and workplaces and cancelling public events were recommended but not mandatory. The only stringent individual policies typically arising in the 31–40 SI range were quarantining high-risk cases from international travel and restrictions on gatherings of 100–1,000 people; however, these two policies are not reflective of citizens’ everyday behavior. Consequently, voluntary behavioral changes appear to be more important drivers of the impact of NPIs on case and death growth rates than are mandatory measures.^3^ This is consistent with other studies that also conclude that the flattening of NPI effectiveness with increasing stringency reflects a relatively stronger voluntary (vs. mandatory) component to behavioral changes (e.g., [17–19, 23]).

**Table 2:** Stringency Index levels and their median constitutent non-pharmaceutical intervention levels

Restriction type	$51-60^{\dagger }$	$41-50^{\diamond }$	$31-40^{\circ }$	Description of $31-40^{\circ }$ levels
school closing	2	1	1	recommend closing
workplace closing	2	1	1	recommend closing (or work from home)
cancellation of public events	2	1	1	recommend cancelling
gatherings restrictions	3	3	2	restrictions on gatherings of 100–1,000 people
transport closing	0	0	0	no measures
stay-home restrictions	1	0	0	no measures
internal mobility restrictions	0	0	0	no measures
international mobility restrictions	3	3	3	quarantine arrivals from high-risk regions
information campaigns	2	2	2	coordinated public information campaign

$^{\dagger }$ upper bound of optimal Stringency Index level for confirmed case growth rate, $^{\diamond }$ upper bound of optimal Stringency Index level for death growth rate, $^{\circ }$ achieves at least 90% of the effectiveness for both case and death growth rates

What causes this voluntary behavioral change? As I have shown, it is partly due to citizens’ expectations of the risk of infection and severity as captured by $\hat{\gamma }_{2}$ in Eq. 3—the effect size, however, was found to be relatively small. The majority of voluntary behavioral change, is likely due to the signalling value of policy decisions. Citizens can use information about the stringency of government measures to infer the severity of the pandemic. This implies that recommendations by governments, for SI ranges of up to 40, were heeded by citizens, who significantly changed their behavior in ways beyond those captured by mobility in Eq. 3, such as by implementing preventative measures including diligent hand washing, wearing masks, and self-isolating when infected.

*Extensive public testing significantly reduces case and death growth rates, contact tracing does not* Figure 5 presents the estimated coefficients and associated 95% confidence intervals regarding contact tracing and public testing (see Table 1 for detailed regression results). All three levels of the testing regime were jointly significantly different from zero ($\chi ^{2}\left( 3\right) =66.03,p<0.0001$), leading to progressively greater declines in case growth rates as testing became more extensive (robust to multiple comparison Sidak corrections).^4^ Note that the most extensive testing policy had an impact of -7.39 [-9.926,-4.854], which is 51% [27%,76%] of that of the most impactful SI range (61–70). Similarly, the implementation of testing policies significantly reduced the death growth rate ($\chi ^{2}\left( 3\right) =42.81,p<0.0001$). Unlike lockdowns, the only costs that testing policies incur are financial; mental health, for example, is generally not affected. Coupled with its significant impact on COVID-19 dynamics, this renders extensive testing a desirable tool.

Contact tracing of any level did not have a significant impact on confirmed case growth rate ($\chi ^{2}\left( 2\right) =1.93,p=0.38$) or death growth rate ($\chi ^{2}\left( 2\right) =1.31,p=0.52$). However, contact tracing may still be effective if the number of daily new cases is small, when efficient tracing is more manageable. There remain important challenges to scaling contact tracing [53–55] that could hinder its effectiveness during significant outbreaks.

*The proportion of the population tested daily does not significantly affect case and death growth rates* While both estimates were negative, as expected, increasing the proportion of daily tests did not significantly reduce the case growth rate, $\hat{\beta }_{5}=-0.18$ [$-0.403,-0.035,p=0.1$], nor did it significantly reduce the death growth rate, -0.126 [$-0.315,0.063,p=0.191$]. Including different levels of the ordinal testing policy variable may partially capture this effect, as they will be correlated to some degree with the proportion tested—that is, extensive testing as coded in the ordinal variable would likely be associated with more testing. Finally, this may be the result of implicitly assuming exogeneity; testing may also be endogenously determined as governments are likely to step up testing during phases with higher transmission.

*Vaccination reduces confirmed case and death growth rates* Despite few datapoints where vaccination was already well underway, each 1 percentage point increase in the cumulative vaccination % reduced the case growth rate by -0.017 percentage points $[-0.032,-0.002,p=0.022]$ and the death growth rate by -0.0187 percentage points [$-0.035,-0.002,p=0.026$]. Note that the median (nonzero) cumulative vaccination % across countries was only 2.6% and the 10th and 90th percentiles ware 0.07% and 19.8%, respectively. Consequently, these relatively low estimates should not be assumed to extrapolate for higher vaccination levels, especially since this is likely to be a nonlinear relationship in reality—that is, the effect of vaccinations may increase at an accelerating rate as the population approaches herd immunity.

*Government policy is endogenous and exhibits hysteresis* Government policy is strongly endogenous, in contrast to the common implicit assumption of exogeneity. The seven-day lagged confirmed growth rate coefficient $\hat{\delta }_{1}=0.048$ $\left[ 0.035,0.061,p<0.0001\right]$ was positively related to NPI severity. Furthermore, I found significant hysteresis in the de-escalation of NPIs. For the same case growth rate, NPIs were significantly more stringent if the case growth rate had recently been falling rather than rising: $\hat{\delta }_{2}=0.531\ [0.453,0.609,p<0.0001]$.

## Discussion and conclusions

A four-equation structural model of multiple agents (SARS-CoV-2 virus, citizens, and governments) capturing the basic dynamics of their endogenous evolution revealed several crucial insights. Recall that the SI maps the stringency of NPI mandates in the range from 0 (no measures taken) to 100. For confirmed case growth rates, there were no significant gains to be had beyond an SI range of 51–60; moreover, 91% of this effect size was achieved with an SI range of 31–40. For death growth rates, no significant gains were to be made beyond an SI range of 41–50, and 93% of this effect size was achieved within the SI range of 31–40. An open testing policy has approximately half the benefits of the optimal NPIs without incurring the societal costs associated with long-term restrictions. Furthermore, the finding that decreases in nonresidential mobility (and therefore increases in time spent at residences) raise the growth rate of confirmed cases and deaths is aligned with contact tracing analyses of heightened transmission risk within a household compared to the wider community [41] and earlier work concluding that shelter-in-place orders did not reduce COVID-19 infection and mortality rates [56].

*Interpretation and implications for policy* Table 2 shows the median values of the individual NPIs measured in the SI for the upper bounds on the socially optimal level of NPI stringency for confirmed case growth rates (51–60) and death growth rates (41–50), as well as the near-optimal range of 31–40. Note that there is significant heterogeneity across NPIs in terms of their severity. The most severe NPI mandates in the three SI ranges featured in Table 2 are restrictions on gatherings and international movement; at the other extreme, no restrictions or recommendations were put in place for transport and internal movements. Stay-home restrictions and recommendations were also mostly absent, with the exception of a recommendation to stay home in the 51–60 range. Moderately severe restrictions were typically implemented in schools and workplaces: The optimal upper bound for confirmed case growth rate includes targeted partial closures of schools and workplaces, whereas the optimal upper bound for death growth rates and the near-optimal SI range (31–40) only feature recommendations to work from home.

These findings are generally aligned with studies finding that more severe restrictions were not significantly more effective than less restrictive policies [1, 17–19]. However, they stand in contrast to others that concluded that strict lockdowns were effective [2, 15] during the first wave, whilst differing in some policy interventions such as school and workplace closures, but agreeing on others such as mass gathering restrictions [2]. Differences between this and other studies may be due to their more limited time series or their lack of explicit behavioral modeling of citizens’ and governments’ reactions to the pandemic. A significant proportion of the effect of NPIs on case and death growth rates can be attributed to voluntary behavioral changes (though often following governmental *recommendations*) rather than mandatory, government-imposed *imperatives*. Notably, the 31–40 range typically included a coordinated public campaign aimed at influencing voluntary behavior. This point highlights the importance of modeling the behavioral incentives of both governments and citizens in conjunction with the pandemic dynamics.

*Strengths and limitations* The primary strength of this study compared to earlier work is its simultaneous modeling of pandemic dynamics with behavioral models of citizens’ adaptation to the pandemic and a model of government policies. This more sophisticated model with behavioral components was made possible by a growth in accumulated data, including testing and vaccination rates. Nonetheless, some simplifications were still necessary to ensure parameter and model identification and to rein in the computational complexity of the estimation processes. These simplifications included examining the effects of countries’ SI levels that are a composite of individual NPIs, rather than examining each NPI separately. Similarly, an average measure of the change in mobility was used instead of disaggregated submeasures of the type of mobility. Furthermore, while random effects allowed for variation across countries in unobservable variables, estimates of the variables of interest (NPIs and other interventions) were pooled across countries. Readers should note that the data did not include the impact of the emergence of the Omicron variant in late 2021. Finally, the dependent variables only reflected the number of confirmed cases and deaths, but did not capture the impact of post-COVID-19 syndrome or “long-COVID”.

*Unanswered questions and further research* As more data become available over time, future research should address some of the limitations of the current modeling. For example, models would ideally allow for heterogeneity in the variable estimates across countries rather than pooling estimates across countries; similarly, subnational data could be used to avoid pooling across regions within countries. Inference about the heterogeneity of NPI effectiveness across different waves and conditional on the predominance of different variants could also be attempted if enough data were available. The analysis of vaccinations should be extended to higher levels of vaccination in order to properly estimate the likely nonlinear effect beyond the low levels reported in this dataset.

Finally, the conclusions of this study must be placed within the context of and validated by other methodological approaches, such as SIR and agent-based models. However, this study offers significant evidence that very stringent NPIs provide no further benefits over moderately stringent ones, and that less stringent NPIs function primarily as signals for significant voluntary changes in citizens’ behavior.

## Supplementary Information

**Additional file 1.** Supplementary Materials.

## Data Availability

Confirmed case and death counts, vaccinations, and tests were download from the COVID-19 Data Hub [35], which also included the implementation of NPIs—a composite score on the Stringency Index—sourced originally from the Oxford COVID-19 Government Response Tracker [37]. Mobility data from the Google Community Mobility Report [38] was merged with the datafile from the Covid-19 Data Hub.

## Abbreviations

NPI: Non-pharmaceutical interventions
SI: Stringency index

## Footnotes

1 Unfortunately, a more rigorous game-theoretic analysis would require significantly more data to effectively infer or observe expectations of agents and the multitude of ways to react to said information.

2 If anything this underestimates the possible range of nonsignificantly different SI ranges, as it ignores the uncertainty associated with whether the range 61–70 is truly the most effective range.

3 I infer that the restriction on gatherings of 100–1,000 people is not the main driver of mandatory behavioral change because the median level of restrictions on gatherings for the next less stringent range, 21–30, is zero, that is, no restrictions whatsoever. Yet the mean estimate of NPI impact for the 31–40 range is -12.945, compared to -10.173 for the 21–30 range. Some of this difference will also be due to other policies that are stricter on average in the former compared to the latter.

4 That is, comparing the baseline of no testing to the first level ($\chi ^{2}\left( 1\right) =12.42,p=0.0013$), the first to the second level ($\chi ^{2}\left( 1\right) =38.55,p<0001$), and the second to the third level ($\chi ^{2}\left( 1\right) =7.83,p=0.0153$).
