# Supplementary material for: On the effectiveness of COVID-19 restrictions and lockdowns: Pan metron ariston
Source: BMC Public Health. 2022 Oct 1;22:1842. doi: 10.1186/s12889-022-14177-7 (PMC9526209; doi:10.1186/s12889-022-14177-7)
Supplement: Supplementary file 1 — Additional file 1. Supplementary Materials. [file 12889_2022_14177_MOESM1_ESM.pdf]

Supplementary material
On the effectiveness of COVID-19 restrictions and lockdowns: Pan metron ariston

Leonidas Spiliopoulos (spiliopoulos@mpib-berlin.mpg.de, corr. author)

17th July 2022

Max Planck Institute for Human Development, Center for Adaptive Rationality
94 Lentzeallee, Berlin 14185, Germany
Phone +49 30 82406-0 (Reception)
Fax +49 30 8249939

Other estimates from the main model

Supplementary Table 1: Other estimates from the structural equation model of $\dot{C}_{i,t}$, $\dot{D}_{i,t}$ $M_{i,t}$ and $SI_{i,t}$

$\dot{C}_{i,t}$		$\dot{D}_{i,t}$	$SI_{i,t}$	
Monday	-0.0198	0.153	α_1^3	-8.621***
	[-0.167,0.128]	[-0.0432,0.349]		[-9.903,-7.340]
Tuesday	0.14	0.189	α_2^3	-6.679***
	[-0.0678,0.347]	[-0.0111,0.389]		[-7.437,-5.920]
Wednesday	0.332***	0.238*	α_3^3	-4.625***
	[0.173,0.491]	[0.0540,0.423]		[-5.252,-3.999]
Thursday	0.278***	0.210*	α_4^3	-3.281***
	[0.145,0.412]	[0.00722,0.413]		[-3.778,-2.784]
Friday	0.392***	0.18	α_5^3	-1.993***
	[0.263,0.521]	[-0.000637,0.361]		[-2.413,-1.574]
Saturday	0.103	0.123	α_6^3	-0.808***
	[-0.00538,0.211]	[-0.0604,0.307]		[-1.192,-0.425]
			α_7^3	0.232
				[-0.131,0.594]
			α_8^3	1.289***
				[0.944,1.635]
			α_9^3	2.617***
				[2.249,2.984]
			α_{10}^3	4.470***
				[4.016,4.924]

Supplementary Table 2: Total effects of SI ranges on the growth rate of confirmed cases

	Coef.	p	95% CI	
1–10	-4.383	0.140	-10.202	1.436
11–20	-5.544	0.012	-9.880	-1.209
21–30	-10.173	0.000	-14.473	-5.874
31–40	-12.945	0.000	-16.883	-9.007
41–50	-13.549	0.000	-17.424	-9.674
51–60	-14.156	0.000	-18.013	-10.298
61–70	-14.426	0.000	-18.319	-10.534
71–80	-14.080	0.000	-17.953	-10.208
81–90	-13.647	0.000	-17.531	-9.763
91–100	-11.958	0.000	-15.906	-8.010

Supplementary Table 3: Difference between the maximum effectiveness level of restrictions for confirmed cases (61–70)

Relative to 61–70	χ^2 (1)	p
1–10	18.310	0.0002
11–20	43.220	0.0000
21–30	21.300	0.0000
31–40	13.910	0.0017
41–50	9.410	0.0193
51–60	2.550	0.6496
71–80	3.480	0.4392
81–90	8.340	0.0343
91–100	43.400	0.0000
Joint	101.710	0.0000

Supplementary Table 4: Total effects of SI ranges on the growth rate of deaths

	Coef.	p	95% CI	
1–10	-7.282	0.000	-10.896	-3.667
11–20	-7.885	0.000	-10.791	-4.979
21–30	-12.344	0.000	-15.047	-9.641
31–40	-14.766	0.000	-17.491	-12.041
41–50	-15.238	0.000	-17.901	-12.576
51–60	-15.541	0.000	-18.175	-12.908
61–70	-15.779	0.000	-18.448	-13.109
71–80	-15.444	0.000	-18.104	-12.785
81–90	-15.267	0.000	-17.968	-12.567
91–100	-14.204	0.000	-16.877	-11.531

Supplementary Table 5: Difference between the maximum effectiveness level of restrictions for deaths (61–70)

Relative to 61–70	χ^2 (1)	p
1–10	25.35	0
11–20	51.57	0
21–30	21.07	0
31–40	10.53	0.0105
41–50	5.79	0.1359
51–60	2.42	0.6832
71–80	4.13	0.3206
81–90	4.3	0.2945
91–100	19.89	0.0001
Joint	81.64	0.0000

Robustness/sensitivity analyses

Construction of the SI index bins

The results below are derived from the alternative modelling assumption that the SI index is binned into quintiles (0, 1–20, 21–40, 41–60, 61–80, 91–100), instead of deciles.

Supplementary Table 6: Generalized structural equation model of $\dot{C}_{i,t}$, $\dot{D}_{i,t}, M_{i,t}$ and $SI_{i,t}$ (binned into quintiles)

$\dot{C}_{i,t}$		$\dot{D}_{i,t}$		$M_{i,t}$		$SI_{i,t}$	
$M_{i,t-7}$	-0.0416***	$M_{i,t-21}$	-0.0173*				
	[-0.0567,-0.0265]		[0.0307,-0.00401]				
$SI_{i,t-14}$		$SI_{i,t-28}$		$SI_{i,t-7}$			
1–20	-5.879**	1–20	-7.939***		-3.566		
	[-10.26,-1.500]		[-10.77,-5.111]		[-10.01,2.880]		
21–40	-12.53***	21–40	-14.05***		-10.50***		
	[-16.70,-8.367]		[-16.72,-11.39]		[-14.81,-6.188]		
41–60	-14.42***	41–60	-15.47***		-15.33***		
	[-18.43,-10.41]		[-18.08,-12.86]		[-19.04,-11.61]		
61–80	-15.19***	61–80	-15.86***		-24.87***		
	[-19.22,-11.16]		[-18.51,-13.21]		[-29.05,-20.69]		
81–100	-14.82***	81–100	-15.55***		-40.06***		
	[-18.89,-10.75]		[-18.24,-12.86]		[-44.92,-35.20]		
$Tpop_{i,t-14}$	-0.18	$Tpop_{i,t-28}$	-0.113	$\dot{C}_{i,t-7}$	-0.244***	$\dot{C}_{i,t-7}$	0.0395***
	[-0.415,0.0552]		[-0.315,0.0890]		[-0.290,-0.198]		[0.0254,0.0536]
$V_{i,t-14}$	-0.0194**	$V_{i,t-28}$	-0.0198*			$I\left[\dot{C}_{i,t-7}\right.$	0.544***
	[-0.0340,-0.00490]		[0.0361,-0.00346]			$\left.-\dot{C}_{i,t-14}\right]$	[0.463,0.625]
$TP_{i,t-14}(1)$	-4.311***	$TP_{i,t-28}(1)$	-2.640**				
	[-6.671,-1.950]		[-4.590,-0.690]				
$TP_{i,t-14}(2)$	-6.482***	$TP_{i,t-28}(2)$	-4.360***				
	[-8.877,-4.087]		[-6.369,-2.351]				
$TP_{i,t-14}(3)$	-7.378***	$TP_{i,t-28}(3)$	-4.977***				
	[-9.821,-4.935]		[-7.077,-2.877]				
$CT_{i,t-14}(1)$	-0.835	$CT_{i,t-28}(1)$	-0.636				
	[-2.043,0.372]		[-1.667,0.395]				
$CT_{i,t-14}(2)$	-0.873	$CT_{i,t-28}(2)$	-0.637				
	[-2.045,0.299]		[-1.669,0.396]				
α^1	21.97***	α^2	20.73***	α^3	3.827*		
	[17.87,26.08]		[18.32,23.14]		[0.356,7.297]		
$\sigma^2_{\lambda,1}$	3.736***	$\sigma^2_{\lambda,2}$	2.537***			$\sigma^2_{\lambda,4}$	3.350***
	[2.442,5.030]		[1.554,3.520]				[2.344,4.355]
$\sigma^2_{\epsilon,1}$	22.48***	$\sigma^2_{\epsilon,2}$	33.91***	$\sigma^2_{\epsilon,3}$	220.9***		
	[18.05,26.91]		[29.57,38.24]		[180.6,261.3]		
$\sigma_{\lambda,12}$=3.001*** [1.948,4.055] , $\sigma_{\lambda,14}$=2.163*** [1.378,2.948] , $\sigma_{\lambda,24}$=1.853*** [1.135,2.572]							
[95% CI],* p<0.05, **p<0.01, *** p<0.001							

Robustness to different COVID-19 waves and SARS-CoV-2 variants

The tables below examine the robustness of the results to alternative restrictions on the date range used for estimation, implicitly varying which waves were included and correspondingly which COVID-19 variants. Table 7 presents the results corresponding to the first wave only (from February 15, 2020 to June 30, 2020). Table 8 presents the results corresponding to the first and second waves, excluding the third wave and the emergence of the Alpha variant (from February 15, 2020 to December 31, 2020).

Supplementary Table 7: Generalized structural equation model of $\dot{C}_{i,t}$, $\dot{D}_{i,t}, M_{i,t}$ and $SI_{i,t}$ (1st wave)

$\dot{C}_{i,t}$		$\dot{D}_{i,t}$		$M_{i,t}$		$SI_{i,t}$	
$M_{i,t-7}$	-0.0108	$M_{i,t-21}$	0.0285*				
	[-0.0361,0.0146]		[0.00491,0.0521]				
$SI_{i,t-14}$		$SI_{i,t-28}$		$SI_{i,t-7}$			
1–10	-0.901		-6.572***	1–10	-0.556		
	[-6.321,4.519]		[-10.21,-2.933]		[-3.975,2.864]		
11–20	-1.2		-5.575***	11–20	-3.856		
	[-5.217,2.817]		[-8.895,-2.255]		[-9.974,2.262]		
21–30	-6.423**		-9.215***	21–30	-6.320***		
	[-10.68,-2.163]		[-12.46,-5.969]		[-9.790,-2.851]		
31–40	-11.06***		-13.43***	31–40	-11.56***		
	[-14.94,-7.189]		[-16.33,-10.53]		[-14.98,-8.140]		
41–50	-11.78***		-13.34***	41–50	-17.64***		
	[-15.52,-8.040]		[-16.28,-10.40]		[-21.00,-14.29]		
51–60	-14.37***		-14.74***	51–60	-19.86***		
	[-17.96,-10.79]		[-17.74,-11.74]		[-23.68,-16.05]		
61–70	-15.19***		-15.77***	61–70	-24.24***		
	[-18.87,-11.51]		[-18.80,-12.74]		[-28.01,-20.46]		
71–80	-15.90***		-15.75***	71–80	-35.02***		
	[-19.60,-12.20]		[-18.73,-12.78]		[-39.44,-30.60]		
81–90	-16.44***		-15.85***	81–90	-41.93***		
	[-20.12,-12.76]		[-18.89,-12.80]		[-45.76,-38.11]		
91–100	-16.89***		-15.62***	91–100	-56.13***		
	[-20.83,-12.95]		[-18.88,-12.36]		[-60.89,-51.36]		
$Tpop_{i,t-14}$	-4.386*	$Tpop_{i,t-28}$	-3.554*	$\dot{C}_{i,t-7}$	-0.104***	$\dot{C}_{i,t-7}$	-0.00158
	[-8.152,-0.620]		[-6.565,-0.543]		[-0.134,-0.0743]		0.00763,0.00448]
$TP_{i,t-14}(1)$	-1.086	$TP_{i,t-28}(1)$	0.67			$I\Big[\dot{C}_{i,t-7}$	0.957***
	[-3.172,1.001]		[-1.422,2.761]			$-\dot{C}_{i,t-14}\Big]$	[0.809,1.106]
$TP_{i,t-14}(2)$	-3.230**	$TP_{i,t-28}(2)$	-0.491				
	[-5.328,-1.132]		[-2.747,1.764]				
$TP_{i,t-14}(3)$	-4.793***	$TP_{i,t-28}(3)$	-1.927				
	[-7.259,-2.327]		[-4.507,0.653]				
$CT_{i,t-14}(1)$	-2.038**	$CT_{i,t-28}(1)$	-1.069				
	[-3.582,-0.494]		[-2.567,0.429]				
$CT_{i,t-14}(2)$	-2.064*	$CT_{i,t-28}(2)$	-1.007				
	[-3.741,-0.387]		[-2.652,0.639]				
α^1	22.60***	α^2	19.84***	α^3	1.495		
	[18.82,26.38]		[17.22,22.46]		[-1.030,4.020]		
$\sigma^2_{\lambda,1}$	8.828***	$\sigma^2_{\lambda,2}$	5.461***			$\sigma^2_{\lambda,4}$	4.968***
	[5.450,12.21]		[3.422,7.500]				[3.237,6.698]
$\sigma^2_{\epsilon,1}$	60.39***	$\sigma^2_{\epsilon,2}$	107.7***	$\sigma^2_{\epsilon,3}$	189.6***		
	[45.00,75.77]		[91.92,123.5]		[154.1,225.2]		

$\sigma_{\lambda,12}$=‘6.251*** [3.734,8.768] , $\sigma_{\lambda,14}$=3.955*** [2.031,5.878] , $\sigma_{\lambda,24}$=2.168** [0.762,3.574]

[95% CI],* $p<0.05$, **$p<0.01$, *** $p<0.001$

Supplementary Table 8: Generalized structural equation model of $\dot{C}_{i,t}$, $\dot{D}_{i,t}, M_{i,t}$ and $SI_{i,t}$ (1st and 2nd waves only)

$\dot{C}_{i,t}$		$\dot{D}_{i,t}$		$M_{i,t}$		$SI_{i,t}$	
$M_{i,t-7}$	-0.0474***	$M_{i,t-21}$	-0.0133*				
	[-0.0526,-0.0423]		[0.0239,-0.00276]				
$SI_{i,t-14}$		$SI_{i,t-28}$		$SI_{i,t-7}$			
1–10	-2.088***		-6.400***	1–10	-2.233		
	[-2.518,-1.658]		[-6.884,-5.915]		[-6.386,1.920]		
11–20	-3.153***		-6.417***	11–20	-4.434**		
	[-3.512,-2.794]		[-6.777,-6.057]		[-7.381,-1.488]		
21–30	-9.721***		-12.09***	21–30	-7.236***		
	[-10.13,-9.310]		[-12.56,-11.61]		[-10.16,-4.308]		
31–40	-12.84***		-14.56***	31–40	-12.28***		
	[-13.29,-12.39]		[-15.17,-13.96]		[-15.31,-9.263]		
41–50	-13.53***		-15.13***	41–50	-13.07***		
	[-13.99,-13.07]		[-15.81,-14.45]		[-16.02,-10.12]		
51–60	-14.33***		-15.50***	51–60	-15.45***		
	[-14.78,-13.87]		[-16.08,-14.93]		[-18.43,-12.47]		
61–70	-15.20***		-15.98***	61–70	-19.50***		
	[-15.70,-14.70]		[-16.59,-15.37]		[-22.46,-16.54]		
71–80	-15.27***		-15.66***	71–80	-29.16***		
	[-15.78,-14.77]		[-16.34,-14.98]		[-32.08,-26.23]		
81–90	-15.29***		-15.72***	81–90	-36.97***		
	[-15.78,-14.79]		[-16.34,-15.10]		[-39.92,-34.02]		
91–100	-14.95***		-15.23***	91–100	-53.80***		
	[-15.50,-14.39]		[-16.09,-14.37]		[-56.76,-50.84]		
$Tpop_{i,t-14}$	-0.562*	$Tpop_{i,t-28}$	-0.482	$\dot{C}_{i,t-7}$	-0.188***	$\dot{C}_{i,t-7}$	0.0119***
	[-1.081,-0.0423]		[-1.708,0.743]		[-0.204,-0.172]		[0.0110,0.0127]
$TP_{i,t-14}(1)$	-4.747***	$TP_{i,t-28}(1)$	-1.973***			$I\Big[\dot{C}_{i,t-7}$	0.396***
	[-4.985,-4.509]		[-2.221,-1.725]			$-\dot{C}_{i,t-14}\Big]$	[0.377,0.414]
$TP_{i,t-14}(2)$	-6.924***	$TP_{i,t-28}(2)$	-3.698***				
	[-7.175,-6.672]		[-4.003,-3.392]				
$TP_{i,t-14}(3)$	-8.083***	$TP_{i,t-28}(3)$	-4.435***				
	[-8.387,-7.778]		[-4.907,-3.962]				
$CT_{i,t-14}(1)$	-1.778***	$CT_{i,t-28}(1)$	-1.070***				
	[-1.954,-1.603]		[-1.361,-0.780]				
$CT_{i,t-14}(2)$	-2.135***	$CT_{i,t-28}(2)$	-1.307***				
	[-2.309,-1.960]		[-1.560,-1.055]				
α^1	23.43***	α^2	20.76***	α^3	2.893		
	[22.13,24.72]		[19.80,21.71]		[-0.0556,5.841]		
$\sigma^2_{\lambda,1}$	6.061**	$\sigma^2_{\lambda,2}$	3.429**			$\sigma^2_{\lambda,4}$	0.879*
	[1.781,10.34]		[0.828,6.030]				[0.143,1.615]
$\sigma^2_{\epsilon,1}$	28.23***	$\sigma^2_{\epsilon,2}$	44.68***	$\sigma^2_{\epsilon,3}$	183.7***		
	[28.14,28.32]		[44.54,44.83]		[183.2,184.2]		
$\sigma_{\lambda,12}$=4.448** [1.191,7.705]‘ , $\sigma_{\lambda,14}$= 1.411 [-0.195,3.018], $\sigma_{\lambda,24}$=1.041 [-0.139,2.22]							
[95% CI],* p<0.05, **p<0.01, *** p<0.001							
